# Evaluation of seven DNA barcodes for differentiating closely related medicinal *Gentiana* species and their adulterants

**DOI:** 10.1186/1749-8546-8-16

**Published:** 2013-08-20

**Authors:** Ka-Lok Wong, Paul Pui-Hay But, Pang-Chui Shaw

**Affiliations:** 1 State Key Laboratory of Phytochemistry and Plant Resources in West China (CUHK), Institute of Chinese Medicine, The Chinese University of Hong Kong, Shatin, Hong Kong, China; 2 School of Life Sciences, The Chinese University of Hong Kong, Shatin, Hong Kong, China

## Abstract

**Background:**

Species identification of living organisms by standard DNA sequences has been well-accepted. Consortium for the Barcode of Life (CBOL) recommends chloroplast regions *rbcL* and *matK* as the DNA barcodes for the land plants. This study aims to evaluate the feasibility and limitations of *rbcL*, *matK*, and 5 other commonly used regions as the DNA barcodes for the medicinal *Gentiana* and their adulterants, *Gentiana. rhodantha* and *Podophyllum hexandrum*.

**Methods:**

The species differentiation power of *rbcL*, *matK*, nuclear internal transcribed spacer (ITS) and 5S rRNA intergenic spacer, and chloroplast *trnH-psbA*, *trnL-F* and *rpl36-rps8* intergenic spacers were tested in different medicinal *Gentiana*, including *Gentiana scabra*, *Gentiana triflora*, *Gentiana manshurica* and *Gentiana rigescens*, from common adulterants such as *Gentiana rhodantha* and *Podophyllum hexandrum* (a toxic herb producing podophyllotoxin).

**Results:**

All seven tested loci could be used to differentiate medicinal *Gentiana* species from their adulterants, and to distinguish *Guanlongdan* from *Jianlongdan*. In terms of general differentiation powers, *rbcL* and *matK* had no significant advantages over the other five loci. Only the 5S rRNA and *trnL-F* intergenic spacers were able to discriminate the closely related species *G. triflora*, *G. scabra* and *G. manshurica.*

**Conclusion:**

The DNA barcodes *rbcL* and *matK* are useful in differentiation of closely related medicinal species of *Gentiana,* but had no significant advantages over the other five tested loci.

## Background

The nuclear and chloroplast genomes are the major targets for plant species authentication and phylogenetic studies. Since the rate of evolution varies across each genome, different DNA regions may be selected to reveal different taxonomic levels. The criteria for a useful DNA marker for authentication are: (1) high interspecific divergence; (2) low intraspecific divergence; (3) short PCR product of around 1 kb; and (4) availability of universal primers for amplification [1,2]. The Consortium for the Barcode of Life (CBOL) set up a standardized sampling method and experimental protocol to analyze agreed-upon “DNA barcodes” [3]. This universal identification system is called DNA barcoding. Recently, the CBOL Plant Working Group recommended that *rbcL* and *matK* should be used as the land plant barcodes [4]. The former offers high universality and good discrimination power, while the latter has higher resolution than other loci. However, it is known that the differentiation powers of *rbcL* and *matK* may not be sufficient for closely related species [5]. Indeed, plenty of land plants are identified by other DNA regions as markers.

The internal transcribed spacer (ITS) of the nuclear ribosomal cistron consists of ITS1 and ITS 2, and has been demonstrated to be useful for phylogenic studies in many angiosperm families [6]. Recently, over 60,000 ITS sequences of plants and animals from GenBank were compared [7]. At the species level, the success rates of identification were 91.9%, 76.1%, 74.2%, 67.1%, 88.1% and 77.4% for animals, dicotyledons, monocotyledons, gymnosperms, ferns and mosses, respectively. ITS regions can be found in plants, animals and fungi, and occasionally ITS regions of fungi in medicinal materials were co-amplified, thereby making direct sequencing of the amplified DNA product unsuccessful. The non-transcribed spacer of 5S rRNA is highly variable, and some studies have illustrated that its resolving power is higher than those of the ITS sequences [8]. In the chloroplast genome, the *trnH*-*psbA* spacer is a rapidly evolving region suitable for identification at the species level [9]. Other chloroplast DNA loci, including *trnL*-*F*, have been demonstrated to be informative at the generic level [10]. In a recent study, *trnL-F* has also been used to separate *Cardiocrinum giganteum* from its variant *C. giganteum* var. *yunnanense* and their closely related species [11].

Four medicinal *Gentiana* species, including *Gentiana manshurica* Kitag., *Gentiana scabra* Bunge, *Gentiana triflora* Pall., and *Gentiana rigescens* Franch., are listed in the Chinese Pharmacopoeia as Gentianae Radix et Rhizoma or “Longdan” in Chinese [12]. They are common medicinal materials used for treating liver diseases [13], and hepatoprotective against acetaminophen-induced acute toxicity [14]. The first three species are mainly distributed in the northeastern part of China and called “*Guanlongdan*” (GL), while *G. rigescens* is located in the southwestern part of China and called “*Jianlongdan*” (JL). The genus *Gentiana* is divided into 12 sections in China [15]. GL and JL belong to the adjacent sections of Pneumonanthe (Section III) and Monopodiae (section IV), respectively. While different plant species may be used for the same medicinal purpose in Chinese medicine (*e.g. Gentiana rhodantha* Franch. is frequently used as a substitute in southwestern China), the neurotoxic *Podophyllum hexandrum* Royle in the family Berberidaceae with a similar morphology is deemed adulterant [16].

This study aims to evaluate the feasibility and limitations of *rbcL* and *matK* and five other commonly used DNA regions for authentication of medicinal *Gentiana* species and their adulterants, *G. rhodantha* and *P. hexandrum*. In particular, the sequence divergences and differentiation powers of the tested regions were determined and compared.

## Methods

Authentic samples were collected from various regions of China, as identified by Dr. Hui Cao based on morphological characters. (Table 1) [17]. The voucher specimens were deposited in the Institute of Chinese Medicine, The Chinese University of Hong Kong.

**Table 1:** Samples studied

**No.**	**Scientific name**	**TCM name**	**Voucher / collection place**	**Voucher**	**GenBank accession number**						
					***rbcL***	***matK***	***trnH-psbA***	***trnL-F***	***rpl36-rps8***	**ITS**	**5S rRNA**
1	*G. manshurica*	Guanlongdan (GL)	Jilin city, Jilin	2005-2701C	JN162107	JN162097	GQ864029	GQ864090	GQ864078	GQ864017	Clone 2: GQ864046
											Clone 3: GQ864047
											Clone 4: GQ864048
2	*G. manshurica*	GL	Dandong, Liaoning	2005-2701D	JN162108	JN162098	GQ864030	GQ864091	GQ864079	GQ864018	Clone 2: GQ864049
											Clone 3: GQ864050
											Clone 4: GQ864051
3	*G. scabra*	GL	Beian, Heilongjiang	2005-2702A	JN162105	JN162093	GQ864027	GQ864088	GQ864076	GQ864015	Clone 1: GQ864039
											Clone 2: GQ864040
											Clone 3: GQ864041
											Clone 4: GQ864042
4	*G. scabra*	GL	Yinan, Heilongjiang	2005-2702B	JN162106	JN162094	GQ864028	GQ864089	GQ864077	GQ864016	Clone 1: GQ864043
											Clone 2: GQ864044
											Clone 4: GQ864045
5	*G. triflora*	GL	Yinan, Heilongjiang	2005-2703A	JN162109	JN162095	GQ864031	GQ864092	GQ864080	GQ864019	Clone 1: GQ864052
											Clone 2: GQ864053
											Clone 13: GQ864054
6	*G. triflora*	GL	Yinan, Heilongjiang	2005-2703B	JN162110	JN162096	GQ864032	GQ864093	GQ864081	GQ864020	Clone 2: GQ864055
											Clone 3: GQ864056
											Clone 4: GQ864057
7	*G. rigescens*	Jianlongdan (JL)	Deqin, Yunnan	2005-2704A	JN162111	JN162099	GQ864033	GQ864094	GQ864082	GQ864021	Clone 1: GQ864058
											Clone 2: GQ864059
											Clone 32: GQ864060
8	*G. rigescens*	JL	Weishan, Yunnan	2005-2704B	JN162112	JN162100	GQ864034	GQ864095	GQ864083	GQ864022	Clone 1: GQ864061
											Clone 2: GQ864062
											Clone 4: GQ864063
9	*G. rhodantha*		Xishui, Guizhou	2005-2706A	JN162113	JN162101	GQ864035	GQ864096	GQ864084	GQ864023	Clone 1: GQ864064
											Clone 3: GQ864065
10	*G. rhodantha*		Shizhu, Chongqing	2005-2706B	JN162114	JN162102	GQ864036	-	GQ864085	GQ864024	Clone 1: GQ864066
											Clone 2: GQ864067
											Clone 3: GQ864068
											Clone 4: GQ864069
11	*P. hexandrum*	Xiaoyelian	Institute of Chinese Medicine, The Chinese University of Hong Kong (ICM-CUHK)	ICM686	JN162115	JN162103	GQ864038	GQ864098	GQ864087	GQ864026	Clone 1: GQ864070
											Clone 2: GQ864071
											Clone 3: GQ864072
12	*P. hexandrum*	Xiaoyelian	Chinese herbal museum, ICM-CUHK	ICM2148	JN162116	JN162104	GQ864037	GQ864097	GQ864086	GQ864025	Clone 1: GQ864073
											Clone 3: GQ864074
											Clone 4: GQ864075

The rhizome of each sample (0.05 g) was ground and total DNA was extracted by a modified CTAB extraction method with a minor modification [18] that the DNA pellet was resuspended in 30 μL of water instead of 50 μL of Tris-EDTA buffer. Polymerase chain reaction was performed in a 25-μL mixture. Details of the primer sequences and the respective amplified regions are presented in Table 2. The specific PCR products were isolated from the PCR mixture by a Gel-M™ Gel Extraction System (Viogene, Taiwan). Except for 5S rRNA, the purified PCR products of the DNA barcodes were directly sequenced. The 5S rRNA PCR product was ligated into the pGEM-T Easy vector (Promega, USA) at 25°C for 2 hours. Three to four clones containing the insert were sequenced for each individual sample. A Rapid Plasmid Miniprep System (Viogene, Taiwan) was used for plasmid extraction. The purified PCR products or plasmids were sequenced using a BigDye® Terminator v3.1 Cycle Sequencing Kit (Applied Biosystems, USA). Alignment of the DNA sequences was accomplished by ClustalW using the BioEdit program [19,20], and manual adjustment of the sequence alignment was performed if necessary. The genetic distance among samples was determined by the nucleotide model Kimura 2-parameter in MEGA 5 software [21]. All distances were calculated from pairwise global alignments, in which alignment gaps and missing data were eliminated by choosing the “pairwise deletion option”. If the minimum sequence divergence between two groups of species was larger than the maximum intraspecific sequence divergence of the two groups of species, the discrimination was considered successful. Phylogenetic trees of the seven loci were constructed by MEGA5 with the neighbor-joining (NJ) method [21]. Bootstrap analyses for 1000 replicates were performed to provide confidence estimates for the tree topologies.

**Table 2:** Universal primers used in this study

**Gene or spacer region**	**Primer name**	**Primer sequence (5′ to 3′)**	**Reference**
*rbcL*	rbcLaF	ATGTCACCACAAACAGAGACTAAAGC	[4]
	rbcLaR	GTAAAATCAAGTCCACCRCG	[4]
*matK*	3 F KIM f	CGTACAGTACTTTTGTGTTTACGAG	[4]
	1R KIM r	ACCCAGTCCATCTGGAAATCTTGGTTC	[4]
*trnH*-*psbA*	trnHf	CGCGCATGGTGGATTCACAATCC	[1]
	psbA3′f	GTTATGCATGAACGTAATGCTC	[1]
*trnL*-*F*	Tab C	CGAAATCGGTAGACGCTACG	[22]
	Tab F	ATTTGAACTGGTGACACGAG	[22]
*rpl36*-*rps8*	rpl36f	CACAAATTTTACGAACGAAG	[1]
	rps8r	TAATGACAGAYCGAGARGCTCGAC	[1]
ITS	ITS5	GGAAGTAAAAGTCGTAACAAGG	[23]
	ITS4	TCCTCCGCTTATTGATATGC	[23]
5S rRNA	S1	GGATCCGTGCTTGGGCGAGAGTAGTA	[24]
	AS1	GGATCCTTAGTGCTGGTATGATCGCA	[24]

## Results

### DNA barcode sequence determination

The primers listed in Table 2 could amplify the corresponding loci in the samples, except for the *trnL-F* region of sample 2005-2706b (*G. rhodantha*) and the *matK* regions of samples 2005-2703b (*G. triflora*), 2005-2704a (*G. rigescens*), 2005-2704b (*G. rigescens*), 2005-2706a (*G. rhodantha*), ICM 686 (*P. hexandrum*) and ICM 2148 (*P. hexandrum*). As a result, we checked the complementarity between the primers and the available *Gentiana* sequences from National Center for Biotechnology information (NCBI). For *matK*, it was found that there are 3–4 different nucleotides between 3 F KIM f and the *Gentiana* sequences (Table 3). We designed a new pair of primers, matK_G SC_F (5′-TATATATTGTATTCGATACAAAC-3′) and matK_GSC_R (5′-TTCTACGAATATTGGAATTGGAA-3′), based on the conserved region of the available *Gentiana* and *P. hexandrum* sequences, which successfully amplified all of the *Gentiana* and *P. hexandrum* samples. For *trnL-F*, there was only one nucleotide difference near the 5′ end terminus of the primer. Since only sample 2005-2706b (*G. rhodantha*) could not be amplified, the failure of amplification might be caused by fragmentation of the template DNA.

**Table 3:** **Sequence alignment among the *matK *forward primer 3 F Kim f and the corresponding binding sites of the *Gentiana *species available in NCBI**

**NCBI Accession no.**	**Scientific name**	**Sequence alignment**
		**3 F Kim f sequence (5′ to 3′)**
		**CGTACAGTACTTTTGTGTTTACGAG**
EF552125.1	*Gentiana acaulis*	....T..A............C..G.
EF552079.1	*Gentiana bavarica*	....T..A............C..G.
EF552115.1	*Gentiana bavarica* subsp. *subacaulis*	....T..A............C..G.
EF552117.1	*Gentiana brachyphylla*	....T..A............C..G.
EF552116.1	*Gentiana brachyphylla*	....T..A............C..G.
EF552100.1	*Gentiana brachyphylla*	....T..A............C..G.
EF552102.1	*Gentiana brachyphylla* subsp. *favratii*	....T..A............C..G.
EF552101.1	*Gentiana brachyphylla* subsp. *favratii*	....T..A............C..G.
EF552124.1	*Gentiana nivalis*	....T..A............C..G.
EF552123.1	*Gentiana nivalis*	....T..A............C..G.
EF552122.1	*Gentiana nivalis*	....T..A............C..G.
EF552121.1	*Gentiana nivalis*	....T..A............C..G.
EF552126.1	*Gentiana prostrata*	....T..A.T..........C..G.
EF552120.1	*Gentiana pumila*	....T..A............C..G.
EF552086.1	*Gentiana pumila* subsp. *delphinensis*	....T..A............C..G.
EF552085.1	*Gentiana pumila* subsp. *delphinensis*	....T..A............C..G.
EF552114.1	*Gentiana rostanii*	....T..A............C..G.
EF552113.1	*Gentiana rostanii*	....T..A............C..G.
EF552078.1	*Gentiana terglouensis*	....T..A............C..G.
EF552107.1	*Gentiana terglouensis* subsp. *schleicheri*	....T..A............C..G.
EF552087.1	*Gentiana terglouensis* subsp. *schleicheri*	....T..A............C..G.
EF552119.1	*Gentiana utriculosa*	....T..A............C..G.
EF552118.1	*Gentiana utriculosa*	....T..A............C..G.
EF552105.1	*Gentiana verna*	....T..A............C..G.
EF552104.1	*Gentiana verna*	....T..A............C..G.
EF552103.1	*Gentiana verna*	....T..A............C..G.
EF552099.1	*Gentiana verna*	....T..A............C..G.
EF552098.1	*Gentiana verna*	....T..A............C..G.
EF552097.1	*Gentiana verna*	....T..A............C..G.
EF552096.1	*Gentiana verna*	....T..A............C..G.
EF552111.1	*Gentiana verna* subsp. *balcanica*	....T..A............C..G.
EF552106.1	*Gentiana verna* subsp. *balcanica*	....T..A............C..G.
EF552112.1	*Gentiana verna* subsp. *pontica*	....T..A............C..G.
EF552110.1	*Gentiana verna* subsp. *pontica*	....T..A............C..G.
EF552109.1	*Gentiana verna* subsp. *pontica*	....T..A............C..G.
EF552108.1	*Gentiana verna* subsp. *tergestina*	....T..A............C..G.

*Gentiana* sequences downloaded from GenBank were converted into their reverse complement before alignment against 3 F Kim f.

### Sequence divergences of the seven DNA regions

The sizes of the seven loci (excluding the primer-binding sites) of the examined species are shown in Table 4. The sizes ranged from 239 to 940 bp, with most falling between 400 to 800 bp as the optimum range for routine PCR. The lengths of the protein-encoding genes *rbcL* and *matK* were identical across the samples, while the five intergenic spacers were found to be varied.

**Table 4:** **Properties of the seven barcoding regions of *Gentiana *and*P. hexandrum***

**Property**	**Species**	***rbcL***	***matK***	***trnH-psbA***	***trnL-F***	***rpl36-rps8***	**ITS**	**5S rRNA**
Average length (bp)	*G. manshurica*	553.0	716.0	482.5	760.5	319.0	693.0	455.3
	*G. scabra*	553.0	716.0	482.5	759.0	319.0	693.0	455.6
	*G. triflora*	553.0	716.0	482.0	762.5	319.0	693.0	456.7
	*G. rigescens*	553.0	716.0	399.0	820.0	317.0	694.0	260.5
	*G. rhodantha*	553.0	716.0	411.0	820.0	304.0	691.0	240.3
	*P. hexandrum*	553.0	716.0	646.0	940.0	524.0	703.5	267.3
GC content (%)	*G. manshurica*	43.8	33.4	24.0	38.7	32.0	58.4	45.7
	*G. scabra*	43.8	33.5	24.0	38.6	32.0	58.6	45.0
	*G. triflora*	43.8	33.3	24.0	38.5	32.0	58.7	46.6
	*G. rigescens*	43.5	33.5	27.5	36.7	30.0	58.4	49.7
	*G. rhodantha*	43.4	33.2	26.5	34.4	32.0	57.6	53.7
	*P. hexandrum*	42.9	34.4	26.0	34.4	37.0	51.0	41.5
Selected polymorphic site*		12233	1125	144	12599	1124	244	12234
		05689	660128	56101	315966	551248	378418	3954472
		666487	137062	840187	895856	066664	560905	9578946
	*G. manshurica*	TCATAA	GTGGAC	TGAATT	ACGCAC	CCCCAG	GTAGCT	CCATGGG
		TCATAA	GTGGAC	TGAATT	ACGCAC	CCCCAG	GTAGCT	CCATGGG
	*G. scabra*	TCATAA	GTGGAC	TGAATT	ACGCGA	CCCCAG	GTAGCT	CCATGGG
		TCATAA	GTGGAC	TGAATT	ACGCGA	CCCCAG	GTAGCT	CCATGGG
	*G. triflora*	TCATAA	GTGGAC	TGAATT	ACGCAC	CCCCAG	GTAGCT	CCGAATC
		TCATAA	GTGGAC	TGAATT	ACGCAC	CCCCAG	GTAGCT	CCGAATC
	*G. rigescens*	TTAAAG	ACGGGG	TGGCAT	ACAGGA	CATTTG	GTGACG	C-G--CT
		TTAAAG	ACGGGG	TGGCAT	ACAGGA	CATTTG	GTGACG	C-G--CT
	*G. rhodantha*	TTGAGA	GTAGGC	TAGAAG	ATATGA	TCCTTC	GCTGTC	CTT--T-
		TTGAGA	GTAGGC	TAGAAG	N/A	TCCTTC	GCTGTC	CTT--T-
	*P. hexandrum*	CTCAGT	TTAAAT	CCGATT	GGAGGA	ACCTAA	ACTGCA	TTA--TT
		CTCAGT	TTAAAT	CCGATT	GGAGGA	ACCTAA	ACTGCA	TTA--TT

*The polymorphic site positions are shown in a vertical manner. For example, the first polymorphic site of *rbcL* is 6, and the second one is 106.

The numbers above the polymorphic sites are their positions in the multiple sequence alignment. ‘–’ in the alignment represents a gap in the DNA sequence.

To show the discriminative powers of the seven DNA regions, we compared the sequence divergence of (1) medicinal *Gentiana* species (*G. scabra*, *G. manshurica*, *G. triflora* and *G. rigescens*) and their adulterants (*G. rhodantha* and *P. hexandrum*); and (2) GL (*G. scabra*, *G. manshurica* and *G. triflora*) and JL (*G. rigescens*) (Table 5). When comparing the divergences between medicinal *Gentiana* species and their adulterants, 5S rRNA had the highest divergence values, both interspecifically and intraspecifically, while *rbcL* had the lowest values (Table 5). The minimum divergence values of *rbcL*, *matK*, *trnH-psbA*, *trnL-F*, *rpl36-rps8*, ITS and 5S rRNA between medicinal *Gentiana* and *P. hexandrum* were 0.0995, 0.3399, 0.3908, 0.3888, 0.2211, 0.4781 and 0.6154, while the maximum intraspecific divergence values were only 0.0018, 0.0042, 0.0101, 0.0026, 0.0033, 0.0058 and 0.0939, respectively. For medicinal *Gentiana* and the adulterant *G. rhodantha*, the minimum divergence values between these regions were 0.0128, 0.0597, 0.1349, 0.1562, 0.0958, 0.0862 and 0.3098, while the maximum intraspecific divergence values were 0.0018, 0.0042, 0.0101, 0.0026, 0.0033, 0.0058 and 0.0914, respectively. Since the maximum intraspecific divergences of the seven loci were lower than the interspecific divergences, all of them could be employed to discriminate between medicinal *Gentiana* species and their adulterants.

**Table 5:** **Sequence divergence of the seven barcoding regions of *Gentiana *and *P. hexandrum***

	***rbcL***	***matK***	***trnH-psbA***	***trnL-F***	***rpl36-rps8***	**ITS**	**5S rRNA**
**(1) Comparison between (A) Medicinal *Gentiana *&(B)*P. hexandrum***							
i) Minimum sequence divergence between Medicinal *Gentiana* &*P. hexandrum*	0.0995	0.3399	0.3908	0.3888	0.2211	0.4781	0.6154
ii) Maximum intraspecific divergence among Medicinal *Gentiana* &*P. hexandrum*	0.0018	0.0042	0.0101	0.0026	0.0033	0.0058	0.0939
iii) Can this barcode distinguish these two groups? ^#^	Yes	Yes	Yes	Yes	Yes	Yes	Yes
iv) Differentiation power ranking^*^	7	5	3	4	6	2	1
**(2) Comparison between (A) Medicinal *Gentiana *&(B)*G. rhodantha***							
i) Minimum sequence divergence between Medicinal *Gentiana* &*G. rhodantha*	0.0128	0.0597	0.1349	0.1562	0.0958	0.0862	0.3098
ii) Maximum intraspecific divergence among Medicinal *Gentiana* &*G. rhodantha*	0.0018	0.0042	0.0101	0.0026	0.0033	0.0058	0.0914
iii) Can this barcode distinguish these two groups? ^#^	Yes	Yes	Yes	Yes	Yes	Yes	Yes
iv) Differentiation power ranking^*^	7	6	3	2	4	5	1
**(3) Comparison between (A) Guanlongdan&(B) Jianlongdan**							
i) Minimum sequence divergence between Guanlongdan & Jianlongdan	0.0109	0.0521	0.0780	0.0332	0.0392	0.0462	0.4897
ii) Maximum intraspecific divergence among Guanlongdan & Jianlongdan	0.0018	0.0042	0.0101	0.0026	0.0000	0.0043	0.0914
iii) Can this barcode distinguish these two groups?^#^	Yes	Yes	Yes	Yes	Yes	Yes	Yes
iv) Differentiation power ranking^*^	7	3	2	6	5	4	1

^#^Barcode is able to distinguish the two groups if (i) > (ii).

*The differentiation power ranking was determined by (i). The most varied locus was ranked as 1 and the least varied locus was ranked as 7.

The DNA sequences were significantly different in GL and JL. The minimum divergence values of *rbcL*, *matK*, *trnH-psbA*, *trnL-F*, *rpl36-rps8*, ITS and 5S rRNA between these two groups were 0.0109, 0.0521, 0.0780, 0.0332, 0.0392, 0.0462 and 0.4897, while the maximum intraspecific divergence values were 0.0018, 0.0042, 0.0101, 0.0026, 0.0000, 0.0043 and 0.0914, respectively. Therefore, GL and JL could be distinguished from each other using any of the seven DNA loci (Table 5). On the other hand, the genetic variability in the three GL species was extremely low for all loci. Only 5S rRNA could differentiate between *G. manshurica* and *G. triflora*, while *trnL-F* could distinguish *G. scabra* and *G. triflora*. Table 4 shows the selected polymorphic sites for differentiating among the three GL species. *G. triflora*, *G. scabra* and *G. manshurica* are genetically closely related, and possess the interchangeable medicinal applications.

To confirm the effectiveness of *rbcL* and *matK* in the identification of *Gentiana* species, we included all available *Gentiana* sequences in NCBI in the analysis of these two barcodes. In total, 14 *rbcL* sequences (including 10 sequences generated in this study) of 9 *Gentiana* species and 68 *matK* sequences (including 10 sequences generated in this study) of 23 *Gentiana* species and subspecies were aligned. For *rbcL*, the maximum intraspecific divergence value was 0.00215, while the minimum interspecific divergence value was 0. We found that the *rbcL* sequences of *Gentiana andrewsii* (HQ590117.1) and *Gentiana pneumonathe* (JN891473.1) were identical. For *matK*, the maximum intraspecific divergence value was 0.01032, while the minimum interspecific divergence value was 0. Twenty sequences were identical, including 12 samples of *Gentiana verna* (EF552088.1–EF552099.1), one sample of *Gentiana schleicheri* (EF552087.1), three samples of *Gentiana pumila* subsp. *delphinensis* (EF552084.1–EF552086.1) and four samples of *Gentiana brachyphylla* subsp. *favratii* (EF552080.1–EF552083.1). These results indicated that *rbcL* and *matK* could not resolve all *Gentiana* species well.

**Figure 1:**
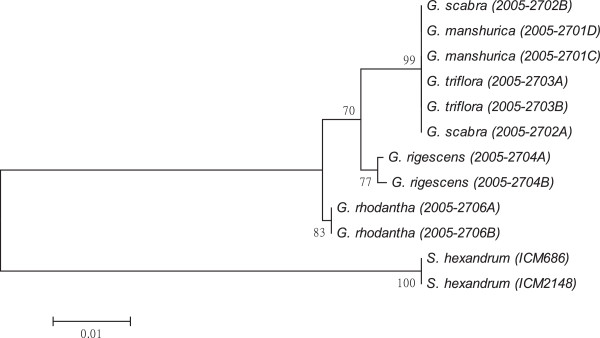
**K2P distance NJ tree for *rbcL.*** A consensus NJ tree for *rbcL* of *Gentiana* and *P. hexandrum* assessed with 1000 bootstrap replicates was constructed by bootstrap analyses with the bootstrap values indicated at the branches (bootstrap values of less than 50 are not shown).

**Figure 2:**
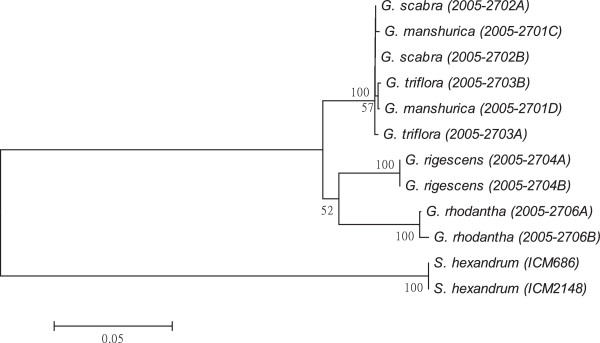
**K2P distance NJ tree for *matK.*** A consensus NJ tree for *matK* of *Gentiana* and *P. hexandrum* assessed with 1000 bootstrap replicates was constructed by bootstrap analyses with the bootstrap values indicated at the branches (bootstrap values of less than 50 are not shown).

**Figure 3:**
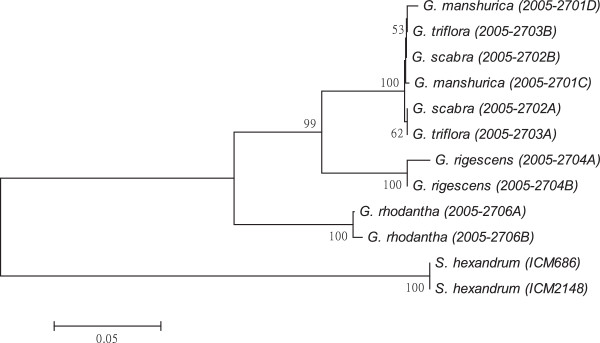
**K2P distance NJ tree for *trnH-psbA.*** A consensus NJ tree for *trnH-psbA* of *Gentiana* and *P. hexandrum* assessed with 1000 bootstrap replicates was constructed by bootstrap analyses with the bootstrap values indicated at the branches (bootstrap values of less than 50 are not shown).

**Figure 4:**
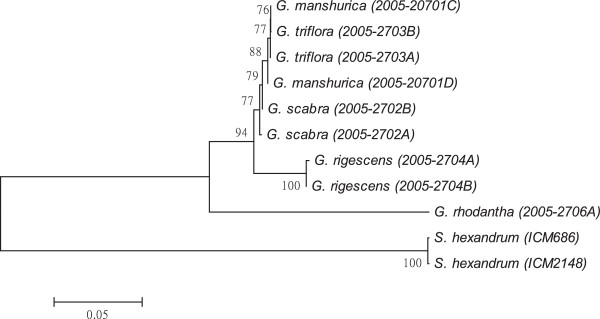
**K2P distance NJ tree for *trnL-F.*** A consensus NJ tree for *trnL-F* of *Gentiana* and *P. hexandrum* assessed with 1000 bootstrap replicates was constructed by bootstrap analyses with the bootstrap values indicated at the branches (bootstrap values of less than 50 are not shown).

**Figure 5:**
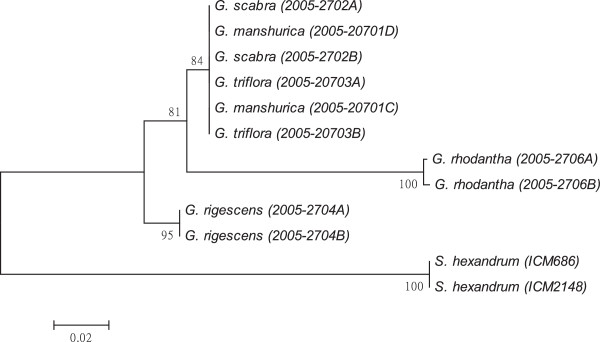
**K2P distance NJ tree for *rpl36-rps8.*** A consensus NJ tree for *rpl36-rps8* of *Gentiana* and *P. hexandrum* assessed with 1000 bootstrap replicates was constructed by bootstrap analyses with the bootstrap values indicated at the branches (bootstrap values of less than 50 are not shown).

**Figure 6:**
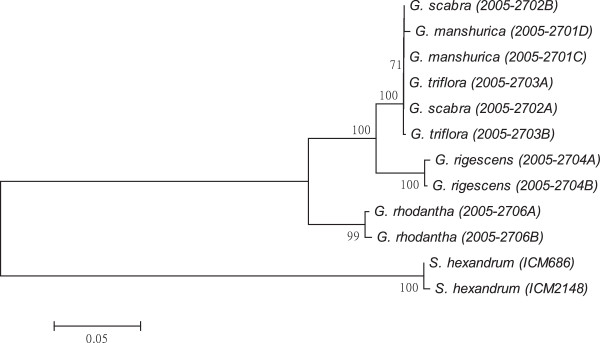
**K2P distance NJ tree for ITS.** A consensus NJ tree for ITS of *Gentiana* and *P. hexandrum* assessed with 1000 bootstrap replicates was constructed by bootstrap analyses with the bootstrap values indicated at the branches (bootstrap values of less than 50 are not shown).

**Figure 7:**
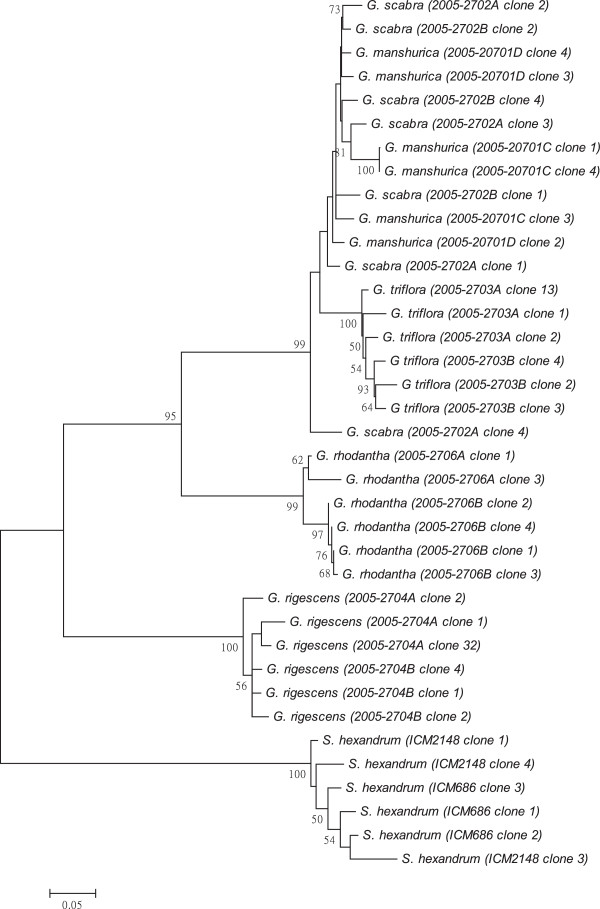
**K2P distance NJ tree for 5S rRNA.** A consensus NJ tree for 5S rRNA of *Gentiana* and *P. hexandrum* assessed with 1000 bootstrap replicates was constructed by bootstrap analyses with the bootstrap values indicated at the branches (bootstrap values of less than 50 are not shown).

As shown in Figures 1, 2, 3, 4, 5, 6 and 7, the NJ trees of the seven barcodes revealed that medicinal *Gentiana* species were clearly differentiated from *P. hexandrum*. Among the *Gentiana* species, the three GL species were clustered together as a clade and separated from JL and *G. rhodantha* with high supporting bootstrap values (>70%), suggesting that the species identification among GL, JL and *G. rhodantha* can be well resolved by the seven DNA barcodes.

## Discussion

This study performed a comparative assessment of the discriminative powers of seven DNA regions for the authentication of genetically closely related medicinal *Gentiana* species and their adulterants. *rbcL* and *matK* are the two recommended DNA barcodes that can resolve 72% of land plants when used in combination [4]. In our study, however, *rbcL* provided the lowest intraspecific and interspecific divergences. There were only 6 bp that differed out of 553 bp between GL and JL. It has also been shown that *rbcL* is the least divergent locus among 11 DNA barcode candidates for differentiating species in Solanaceae [1].

The other CBOL-recommended barcode *matK* had higher sequence divergence, but was difficult to amplify by PCR. There were mismatches between the primer and the published *Gentiana* sequences, indicating that the recommended *matK* primers might not be applicable to all land plants. A recent study of medicinal plants in Southern Morocco [25] shows that the success rate of PCR amplification of *matK* is less than 30%. Regarding the resolving power, *matK* had the third-highest value for differentiating between GL and JL (Table 5). Nevertheless, it was only ranked fifth and sixth for distinguishing between medicinal *Gentiana* species and their adulterants *P. hexandrum* and *G. rhodantha*, respectively.

*trnL*-*F* had the longest DNA sequence among the tested loci (Table 4). A *Gentiana* sample could not be amplified, which was probably due to fragmentation of the DNA or other reasons. *trnL-F* had a high resolving power, and was the only locus capable of differentiating *G. scabra* from *G. triflora* (Table 4), suggesting *trnL-F* as a good locus for differentiation of the closely related *Gentiana* species.

The size of *rpl36*-*rps8* was small among the seven loci (Table 4) The PCR product of *P. hexandrum* was about 200 bp larger than those of *Gentiana.* Thus, the size difference could be used as a marker to distinguish *Gentiana* from *P. hexandrum* without DNA sequencing. Like *rbcL*, *rpl*36-*rps*8 also had low interspecific and intraspecific divergences, although its ranking was slightly higher than that of *rbcL*. Its major drawback was the limited number of reference sequences in GenBank.

The size of the *trnH*-*psbA* region ranged from 399 to 646 bp, which was moderate among the seven DNA regions (Table 4). There was a significant size difference between *Gentiana* and *Podophyllum*. In terms of the resolving power, *trnH-psbA* had ranked second for differentiating GL from JL, and provided higher resolving power than *matK* and *rbcL*. This intergenic spacer also shows a good amplification success rate and discrimination power among the nine loci tested [1]. Among 19 species in seven families of angiosperms, *trnH*-*psbA* shows nearly three-fold higher divergence than other tested chloroplast regions, while the ITS region exhibits two-fold higher divergence than *trnH*-*psbA*[1].

Some studies [26-28] show that nuclear ITS is an appropriate DNA marker for herbal authentication and plant phylogenetic studies. In our study, the ITS region was the third longest region across *Gentiana* and *P. hexandrum*, and the sizes differed slightly from one another (Table 4). The divergence ranking was average among the five *Gentiana* species, but increased to the second highest for distinguishing medicinal *Gentiana* and *P. hexandrum* (Table 5), indicating that the ITS regions among the studied *Gentiana* species were quite conserved.

The size of the 5S rRNA intergenic spacer regions ranged from 239 to 457 bp, which was the smallest but most varied (Table 4). Among the tested regions, only 5S rRNA could distinguish *G. triflora* from *G. manshurica* and *G. scabra.* Our study showed that the intraspecific divergence was high, which was probably due to the non-homogeneity of the different copies of the 5S rRNA gene spacer. It is essential to clone the amplified PCR product prior to sequencing to overcome the sequence degeneration issue.

Jiang *et al.*[29] established chemical profiles of *Gentiana* species. The four medicinal *Gentiana* species involved have close similarity in their chemical compositions, in that they all contain loganic acid, 6-O-B-D-glucopyranosylgentiopicroside, swertiamarin, gentiopicroside, and sweroside [29]. Compound 2-(o,m-dihydroxybenzyl)-sweroside is only found in *G. rigescens*[29] and can be used to differentiate GL and JL. Among the three GL species, only *G. triflora* contains gentiotrifloroside [29]. The chemical profiles of *G. manshurica* and *G. scabra* are nearly identical, except that the former has a higher sweroside content [29]. The chemical profiles therefore support our observations in the DNA barcode analyses.

## Conclusion

All the tested loci could differentiate medicinal *Gentiana* species from their adulterants, and distinguish GL from JL. The two official DNA barcodes, *rbcL* and *matK*, have no significant advantages over the remaining five loci examined.

## Abbreviations

CBOL: Consortium for the Barcode of Life; GL: Guanlongdan; JL: Jianlongdan; NCBI: National Center for Biotechnology Information.

## Competing interests

The authors declare that they have no competing interests.

## Authors’ contributions

KLW generated the DNA barcode sequences and performed data analyses. PPHB collected herbal materials and designed the study. PCS coordinated the study. KLW and PCS wrote the manuscript. All authors read and approved the final manuscript.
